# Physical–MAC Layer Integration: A Cross-Layer Sensing Method for Mobile UHF RFID Robot Reading States Based on MLR-OLS and Random Forest

**DOI:** 10.3390/s26020491

**Published:** 2026-01-12

**Authors:** Ruoyu Pan, Bo Qin, Jiaqi Liu, Huawei Gou, Xinyi Liu, Honggang Wang, Yurun Zhou

**Affiliations:** College of Communication and Information Engineering, Xi’an University of Posts and Telecommunications, Xi’an 710121, China; panruoyu@xupt.edu.cn (R.P.); liujiqi@stu.xupt.edu.cn (J.L.); gouhuawei@stu.xupt.edu.cn (H.G.); lxy829@stu.xupt.edu.cn (X.L.); wanghonggang@xupt.edu.cn (H.W.); zhouyurun@stu.xupt.edu.cn (Y.Z.)

**Keywords:** UHF RFID, mobile robot, shelf positioning, goods distribution sensing, adaptive reading

## Abstract

In automated warehousing scenarios, mobile UHF RFID robots typically operate along preset fixed paths to collect basic information from goods tags. They lack the ability to perceive shelf layouts and goods distribution, leading to problems such as missing reads and low inventory efficiency. To address this issue, this paper proposes a cross-layer sensing method for mobile UHF RFID robot reading states based on multiple linear regression-orthogonal least squares (MLR-OLS) and random forest. For shelf state sensing, a position sensing model is constructed based on the physical layer, and MLR-OLS is used to estimate shelf positions and interaction time. For good state sensing, combining physical layer and MAC layer features, a K-means-based tag density classification method and a missing tag count estimation algorithm based on frame states and random forest are proposed to realize the estimation of goods distribution and the number of missing goods. On this basis, according to the read state sensing results, this paper further proposes an adaptive reading strategy for RFID robots to perform targeted reading on missing goods. Experimental results show that when the robot is moving at medium and low speeds, the proposed method can achieve centimeter-level shelf positioning accuracy and exhibit high reliability in goods distribution sensing and missing goods count estimation, and the adaptive reading strategy can significantly improve the goods read rate. This paper realizes cross-layer sensing and read optimization of the RFID robot system, providing a theoretical basis and technical route for the application of mobile UHF RFID robot systems.

## 1. Introduction

In recent years, Radio Frequency Identification (RFID) technology, especially in the Ultra-High Frequency (UHF) band, has been widely applied across various industries due to its advantages such as non-line-of-sight reading capability, rapid batch identification, and cost-effectiveness [1,2,3]. Particularly in industrial and commercial environments like automated warehouses, smart retail, and intelligent archives [4,5], integrating UHF RFID readers onto mobile robot platforms further enhances their practicality, offering flexibility for large-scale inventorying and overcoming the inherent blind spots of static reader deployments [6,7]. However, since the signal backscattered from RFID tags is relatively weak and susceptible to interference from various environmental factors in the transmission environment [8]. The backscattered signal of the tag naturally carries the characteristics of the environmental factors in the transmission environment, which promotes the transformation of the RFID system from the “identification” function to the “sensing” function. Therefore, by utilizing the characteristic of the RFID backscatter communication mechanism being sensitive to the transmission environment, the state of the sensing tag can be perceived based on the variation characteristics of various environmental factors carried in the tag’s reflected signal [9,10].

However, there are still great challenges in moving from simply collecting basic information of tags to achieving comprehensive sensing of tag states [11,12]. In practical warehousing applications, mobile RFID robot systems usually operate along preset fixed paths and have two fundamental problems. Firstly, mobile robots lack the autonomous sensing ability of environmental states during interaction with shelves, and the execution of tasks relies on preset shelf position information [13]. Once the shelf layout changes, the system often cannot automatically recognize it and must be recalibrated through human intervention [14]. Secondly, the inventory process is usually limited to the collection of basic tag information, and it is impossible to obtain more complex state information such as the distribution of items on shelves, stacking density, and potential occlusion. Therefore, when facing scenarios such as dense tags or severe occlusion, it is difficult to timely detect and compensate for missing reads, thereby affecting the integrity and accuracy of the overall inventory data [15,16].

To address the above limitations, this paper proposes a cross-layer sensing method for mobile UHF RFID robot reading states based on MLR-OLS and random forest. This system not only collects basic tag information but also fully utilizes the characteristic information of RFID signals to perceive the interaction state between the robot and shelves, as well as the status of goods. This paper proposes two models: a position sensing model of robot-shelf interaction and a distribution sensing model of goods tags. The position sensing model of robot-shelf interaction uses physical layer features of RFID signals to calculate the shelf’s coordinate position and the precise moments when the robot enters or leaves the shelf area. The distribution sensing model of goods tags obtains the distribution of goods on the shelves by analyzing communication mechanisms such as energy competition and MAC layer collisions, and estimates the number of goods that are not read. This helps to identify potential anomalies during the inventory process, providing a basis for subsequent design of adaptive inventory strategies, and improving the reading accuracy and inventory efficiency of RFID robot systems [17,18].

The main contributions of this paper are summarized as follows:

1. A position sensing model of robot-shelf interaction is proposed, which utilizes tag characteristic values to calculate the shelf’s position coordinates.
2. A time estimation algorithm based on MLR-OLS is designed to detect key moments when the robot enters and leaves the shelf area, ensuring that the RFID robot system can perform goods inventory within the optimal tag reading area.
3. Analyze the changes in tag feature values under different tag density conditions, construct a distribution sensing model of goods tags, and design a K-means-based goods tag density classification method to perceive the distribution of goods tags on shelf.
4. Design a missing tag count estimation algorithm based on frame states and random forest. Use observable MAC layer statistical data (such as collision, idle, and successful time slots) to estimate the number of missing tags.
5. The research results of this paper provide support for the design of subsequent adaptive inventory strategies, contributing to the improvement of reading accuracy and inventory efficiency of RFID robot systems in dynamic inventory scenarios such as unmanned warehousing and smart retail.

The remainder of this paper is organized as follows: Section 2 introduces related work. Section 3 presents the system design. Section 4 constructs a position sensing model of robot-shelf interaction. Section 5 constructs the distribution sensing model of goods tags. Section 6 describes the experimental setup and details of experimental verification. Section 7 concludes the paper and discusses future work.

## 2. Related Work

This section will primarily describe existing research and relevant technical methods in two aspects: first, the integration of RFID technology with mobile robots to achieve dynamic environmental sensing; second, it is estimated that the number of tags failed to be read successfully in RFID inventory.

### 2.1. Dynamic Sensing in RFID Robotic Systems

Integrating RFID technology into mobile robots aims to break through the coverage limitations of fixed readers, enabling RFID systems to move autonomously in a wide space and collect tag data. Early research mainly focused on using the physical layer characteristics of RFID signals for high-precision positioning. For example, DiGiampaolo and Martinelli [19] proposed a two-step method for positioning items on shelves: first, the robot achieves self-positioning using a small number of reference tags and a Kalman filter; based on the known trajectory, it uses the collected phase of item tags to resolve the position of items on shelves through an electromagnetic model. Tripicchio et al. [20] adopted the idea of Synthetic Aperture Radar (SAR) and realized accurate estimation of the two-dimensional coordinates of tags by processing the phase information of tags collected by the robot during movement combined with a hyperbolic algorithm. Later, the research focus shifted from simple coordinate positioning to more complex dynamic scene sensing. The work of Wu et al. [21] is a typical representative of this direction; by utilizing the phase difference between two antennas, the robot can effectively maintain the relative attitude with dynamic targets without pre-constructing an environmental model or performing precise calibration. Xie et al. [22] proposed deploying dual tags on target goods in shelf scenarios, and resolving the three-dimensional position of the target on the shelf by analyzing the phase difference between the dual tags. Although existing studies have made significant progress in robot navigation and tag positioning, there is still insufficient attention to the dynamically changing relative positional relationship between shelves and robots during the inventory process. A key prerequisite for ensuring the reliability of tag data reading is that the robot needs to find the optimal reading area of goods tags for inventory.

### 2.2. Tag Quantity Estimation

In practical goods inventory using RFID technology, not all tags can be successfully read due to factors such as channel collisions, signal attenuation, and tag occlusion. Therefore, estimating the number of missing tags is an important research content of RFID sensing technology. Researchers usually start with protocols; Xi et al. [23] extended the traditional static tag count estimation problem to dynamic scenarios and designed a set of protocols SSR/ESSR to estimate the number of lost tags that left the system and unknown tags that newly entered the system within a time window. Chen et al. [24] designed a two-phase protocol, ERMI: first, actively activate unknown tags in the system to eliminate interference, then identify lost tags in the updated environment. The EUMD protocol proposed by Lin et al. [25] also activates unknown tags first and then uses optimized hash functions to improve the reliability of detecting lost tags. Although the above protocol design methods are ingenious, they often require multiple rounds of interaction and are relatively complex to implement. Another type of research focuses on estimating the number of tag groups.

Wang et al. [26] studied the cardinality estimation problem of unknown tags and proposed a tag count estimation scheme, which estimates the number of tags by utilizing the feature of the number of idle time slots in the prediction frame composed of known tags transforming into non-idle time slots in the actual response frame. Su et al. [27] considered the fundamental impact of unreliable channels on the ALOHA protocol, re-derived the probabilities of collision, idle, and successful time slots by establishing a probabilistic identification model, and proposed a cardinality estimation algorithm that can adapt to channel changes on this basis. Most of the above studies rely on probabilistic modeling and analysis of communication protocols, in recent years, the research trend has been to directly estimate the number of tags using tag data through machine learning methods. Dujić Rodić et al. [28] transformed the total tag count estimation problem into a multi-class classification task and systematically evaluated the feasibility of various machine learning classifiers using time slot statistical information to infer the total number of tags. This paper draws on and advances this idea to estimate the number of missing tags using MAC layer statistics based on the frame-slotted ALOHA protocol.

## 3. System Design

**Figure 1:**
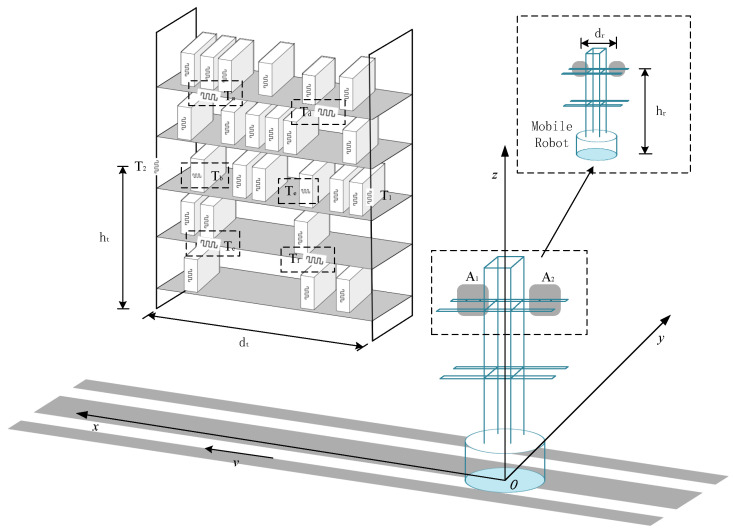
Schematic diagram of RFID robot system architecture.

The architecture of the RFID robot system is shown in Figure 1. Eight RFID tags are pasted at fixed positions on the shelf, and the tag definition is shown in Table 1; shelf localization tags are attached to the pillars on the left and right sides of the shelf, with the distance between tags being the length of the shelf $d_{t}$, and the height being $h_{t}$. Shelf reference tags are affixed within six different regions of the shelf, goods to be tested are randomly deployed on the shelf, with each goods carrying an RFID tag.

In addition, the RFID robot is equipped with a portable computer and an RFID reader. The reader is connected to two directional antennas, denoted as $A_{1}$ and $A_{2}$. Both antennas are oriented towards the shelf and are vertically mounted on the robot. The distance between $A_{1}$ and $A_{2}$ is $d_{r}$, and their installation height is $h_{r}$. This robot is designated as the RFID Robot.

When the RFID robot system is running, the robot moves at a constant speed *v*, with its direction of movement parallel to the shelf. In this article, a spatial rectangular coordinate system is established with the projection of the robot’s starting position’s center of gravity on the ground as the coordinate origin *O*, and the directions of each coordinate axis are shown in Table 2. The starting coordinates of antennas $A_{1}$ and $A_{2}$ are, respectively, $\left(\frac{1}{2}d_{r},0,h_{r}\right)$ and $\left(-\frac{1}{2}d_{r},0,h_{r}\right)$. The RFID robot forwards the collected tag data (EPC, RSSI, phase, antenna port, timestamp, inventory count, etc.) to the portable computer through an Ethernet connection for data storage and processing. As the RFID robot moves forward, when the dual antennas collect the signals of the shelf positioning tags $T_{1}$ or $T_{2}$, the processed data is brought into the position sensing model of robot-shelf interaction, and the position coordinates of the shelf are obtained in combination with the robot’s positioning system, and the behavior of the RFID robot entering and leaving the shelf is judged.

After the RFID robot enters the optimal reading area of goods tags, it waits to move to the optimal reading area for tag reading. The RFID data of the read shelf reference tags and goods tags are brought into the distribution sensing model of goods tags after operations such as preprocessing and feature extraction to evaluate the goods density. Based on the density difference, the shelf area is divided into goods missing read risk areas of different levels, the number of missing read goods is estimated.Finally, an adaptive reading strategy for the RFID robot is designed according to the sensing results.

**Figure 2:**
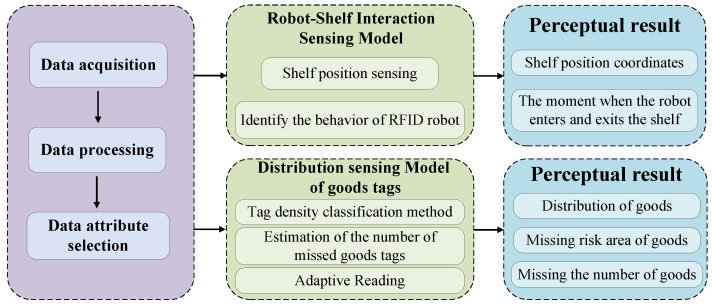
Flowchart of RFID robot system.

In this paper, the system flow chart of Physical–MAC layer integration, a cross-layer sensing method for mobile UHF RFID robot reading states based on MLR-OLS and random forest, is shown in Figure 2. It includes data processing, a position-sensing model of robot-shelf interaction, and a distribution-sensing model of goods tags. In the position sensing model of robot-shelf interaction, this paper constructs a spatial coordinate system for the position sensing model, combines the geometric relationships in the coordinate system with the tag characteristic values collected by the antenna to achieve shelf position sensing and obtain the position coordinates of the shelf, and on this basis adopts the MLR-OLS algorithm to obtain the time points when the RFID robot enters and exits the shelf. In the distribution sensing model of goods tags, the K-means algorithm is utilized to perform tag density clustering according to the tag characteristic values, and the goods areas with different densities are further divided into goods missing-reading risk areas of different levels; on this basis, the random forest algorithm is applied to estimate the number of missed goods tags, and finally adaptive identification of goods tags is realized according to the sensing results.

## 4. Position Sensing Model of Robot-Shelf Interaction

This section elaborates on the position sensing model of robot-shelf interaction in detail. A spatial coordinate system is established based on the indoor laser positioning system, and the position sensing model is used to obtain the shelf position and identify the behavior of the RFID robot entering and leaving the shelf.

### 4.1. Shelf Position Sensing

**Figure 3:**
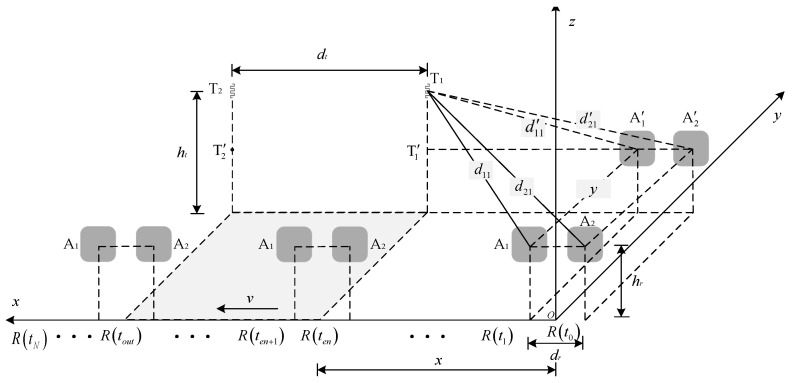
The spatial coordinate system of the position sensing model of robot-shelf interaction.

The spatial coordinate system of the position sensing model of robot-shelf interaction is shown in Figure 3. When the antenna radiation direction is perpendicular to the tag plane, the recognition performance of the RFID system is optimal. Therefore, the shaded area on the ground is represented as the optimal reading area for goods tags.

Suppose that the sampling frequency of the entire operation process of the robot system is *N*. Let $R\left(t_{n}\right)=\left(x,y,z\right)$ represent the position coordinates of the robot at time $t_{n}\left(n\in \left[0,N\right]\right)$. The matrix of the robot’s position *R* can be expressed as

(1) $$\begin{array}{cc} R(t_{n}) & =[R\left(t_{0}\right),\cdots ,R\left(t_{en}\right),\cdots ,R\left(t_{out}\right),\cdots ,R\left(t_{N}\right)]\\ \; & =[\left(0,0,h_{r}\right),\cdots ,\left(vt_{en},0,h_{r}\right),\cdots ,\left(vt_{out},0,h_{r}\right),\cdots ,\left(vt_{N},0,h_{r}\right)] \end{array}$$

where, en∈(0,N), out∈(0,N), and en<out. Divide the operation process of the RFID robot system into three stages: before entering the optimal reading area $\left[R\left(t_{0}\right),R\left(t_{en}\right)\right]$, within the optimal reading area $\left[R\left(t_{en}\right),R\left(t_{out}\right)\right]$, and after leaving the optimal reading area $\left[R\left(t_{out}\right),R\left(t_{N}\right)\right]$.

In order to facilitate calculation, the antennas are denoted as $A_{i}$ (i=1,2), and the shelf positioning tags are denoted as $T_{j}$ (j=1,2). The antennas $A_{1}$ and $A_{2}$ are horizontally projected onto the same plane as the shelf positioning tags, with their projection points denoted as $A_{1}^{\prime }$ and $A_{2}^{\prime }$, respectively. Let $d_{ij}^{\prime }\left(t_{n}\right)$ represent the distance between the antenna projection point $A_{i}^{\prime }$ and the tag $T_{j}$. A perpendicular line is drawn from tag $T_{j}$ to the horizontal plane xoy, intersecting the extension of $\vec{A_{2}^{\prime }A_{1}^{\prime }}$ at point $T_{j}^{\prime }$. The spatial coordinates of tag $T_{j}$ are defined as $C_{j}=\left[x_{j},y_{j},h_{t}\right]$, and according to the geometric relationship of the spatial coordinate system, for tag $T_{1}$ before the RFID robot enters the optimal reading area, it can be concluded that

(2) $$\left\{\begin{array}{c} d_{11}^{2}\left(t_{n}\right)=d_{11}^{\prime 2}\left(t_{n}\right)+y^{2}\left(t_{n}\right)\\ d_{11}^{\prime 2}\left(t_{n}\right)=x_{11}^{2}\left(t_{n}\right)+{\left(h_{t}-h_{r}\right)}^{2} \end{array}\right.\;\left\{\begin{array}{c} d_{21}^{2}\left(t_{n}\right)=d_{21}^{\prime 2}\left(t_{n}\right)+y^{2}\left(t_{n}\right)\\ d_{21}^{\prime 2}\left(t_{n}\right)=x_{21}^{2}\left(t_{n}\right)+{\left(h_{t}-h_{r}\right)}^{2} \end{array}\right.$$

Further:

(3) $$\left\{\begin{array}{c} d_{11}^{2}\left(t_{n}\right)-{\left(h_{t}-h_{r}\right)}^{2}=x_{11}^{2}\left(t_{n}\right)+y^{2}\left(t_{n}\right)\\ d_{21}^{2}\left(t_{n}\right)-{\left(h_{t}-h_{r}\right)}^{2}=x_{21}^{2}\left(t_{n}\right)+y^{2}\left(t_{n}\right) \end{array}\right.$$

Here, the distance between antenna $A_{i}$ and tag $T_{j}$ is denoted as $d_{ij}\left(t_{n}\right)$,$x_{ij}\left(t_{n}\right)$ represents the horizontal distance between tag $T_{j}$ and the robot in the x-axis direction calculated from the data collected by antenna $A_{i}$ at time $t_{n}$, and $y(t_{n})$ represents the coordinate of tag $T_{1}$ in the y-axis direction. Define the phase matrix of the collected shelf positioning tags as

(4) $$\Theta =\left[\begin{array}{c} \Theta_{11},\Theta_{21},\Theta_{12},\Theta_{22} \end{array}\right]$$

Further:

(5) $$\Theta_{ij}\left(t_{n}\right)=\left[\Theta_{ij}\left(t_{0}\right),\cdots ,\Theta_{ij}\left(t_{en}\right),\cdots ,\Theta_{ij}\left(t_{\mathrm{out}}\right),\cdots ,\Theta_{ij}\left(t_{N}\right)\right]$$

Here, Θ represents the phase value collected by the reader, where i=1,2 corresponds to antennas $A_{1}$ and $A_{2}$, and j=1,2 corresponds to the shelf reference tags $T_{1}$ and $T_{2}$. The phase information obtained in RFID communication can reflect changes in the distance between the tag and the reader. Theoretically, the phase exhibits a periodic distribution within the range [0,2π). However, in practical scenarios, factors such as multipath reflection cause the measured phase values to exceed periodic boundaries, resulting in discontinuities. Therefore, phase unwrapping is required to restore phase continuity and eliminate ambiguities caused by periodicity. The mathematical expression of the measured phase is

(6) $$\left\{\begin{array}{c} \Theta_{ij}\left(t_{n}\right)=\;\mathrm{mod}\;\left[\theta_{ij}\left(t_{n}\right)+\phi ,2\pi \right]\\ \theta_{ij}\left(t_{n}\right)=2\pi \frac{2d}{\lambda }\\ \phi =\phi_{d}+\phi_{e} \end{array}\right.$$

Here, θ represents the cumulative phase of signal propagation, while ϕ denotes the aggregate phase offset comprising hardware-induced phase shift $\phi_{d}$ and environment-induced phase shift $\phi_{e}$. The measured value Θ is the result of taking the modulo 2π of the total phase shift accumulated from all phase signals received by the reader.

**Figure 4:**
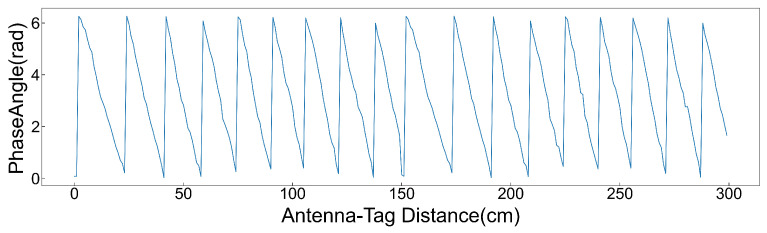
Schematic diagram of the relationship between phase and distance.

In order to verify the relationship between the original phase and the wrapped phase, this paper tests it in a darkroom environment. Under controlled conditions with all other factors constant, the antenna was fixed while the tag was moved away incrementally. Phase data were collected every 5 cm, with each measurement lasting 30 s, and the average phase was recorded. The results, shown in Figure 4, indicate that as the distance between the tag and antenna increases, the tag’s phase exhibits stable periodic behavior within the range [0,2π), consistently remaining in this interval.

**Figure 5:**
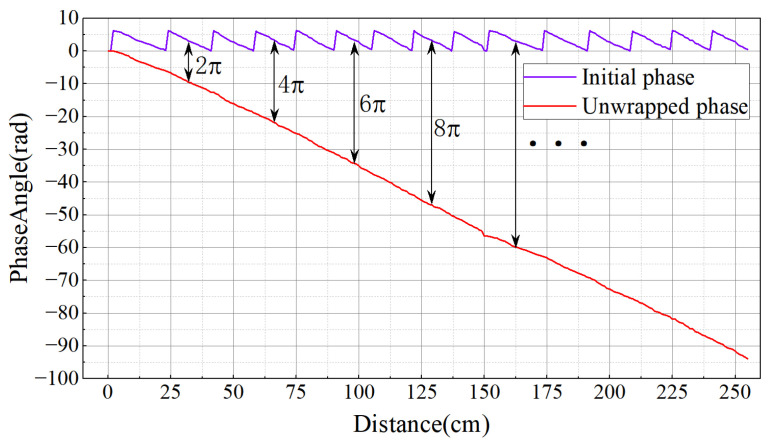
Schematic diagram of the relationship between unwrapped phase and distance.

In this paper, a treatment similar to [29] is used to eliminate the phase jump phenomenon. Since continuous phase values do not exhibit abrupt jumps, phase wrapping can be detected by evaluating the difference between adjacent data points. Each point is then unwrapped by adding an integer multiple of 2π. As shown in Figure 5, the unwrapped phase demonstrates a strong linear relationship with distance. The unwrapped phase can be expressed as

(7) $$\theta \left(t_{n}\right)=\Theta \left(t_{n}\right)+2k\pi \;\left(k=1,2,\cdots \right)$$

The analytical expression of the phase matrix established after phase unwrapping for the *N* groups of phase data continuously collected during the robot’s operation is

(8) $$\theta =\left[\theta_{11},\theta_{21},\theta_{12},\theta_{22}\right]$$

Further:

(9) $$\theta_{ij}\left(t_{n}\right)=\left[\theta_{ij}\left(t_{0}\right),\cdots ,\theta_{ij}\left(t_{en}\right),\cdots ,\theta_{ij}\left(t_{\mathrm{out}}\right),\cdots ,\theta_{ij}\left(t_{N}\right)\right]$$

After obtaining the expanded phase matrix, according to the phase-based ranging formula,

(10) $$d_{ij}\left(t_{n}\right)=\frac{\lambda \theta_{ij}\left(t_{n}\right)}{4\pi }$$

the distance $d_{ij}\left(t_{n}\right)$ from antenna $A_{i}$ to tag $T_{j}$ at time $t_{n}$ can be obtained, where $\theta_{ij}\left(t_{n}\right)$ represents the theoretical phase value read by antenna $A_{i}$ from tag $T_{j}$ at time $t_{n}$, and λ denotes the wavelength of the electromagnetic wave emitted by the reader. Therefore, at time $t_{n}$, the distance *d* between antenna $A_{i}$ and tag $T_{j}$ can be expressed as

(11) $$d=\left[d_{11},d_{12},d_{21},d_{22}\right]$$

Further,

(12) $$d_{ij}\left(t_{n}\right)=\left[d_{ij}\left(t_{0}\right),\cdots ,d_{ij}\left(t_{n}\right),\cdots ,d_{ij}\left(t_{N}\right)\right]$$

From the known relationships, it can be obtained that

(13) $$|x_{2j}\left(t_{n}\right)-x_{1j}\left(t_{n}\right)|=d_{r},\;R\left(t_{n}\right)\in \left[R\left(t_{0}\right),R\left(t_{en}\right)\right]$$

That is, before the RFID robot enters the optimal reading area, the x-axis coordinate of tag $T_{j}$ measured by antenna $A_{2}$ differs from the x-axis coordinate of tag $T_{j}$ measured by antenna $A_{1}$ by $d_{r}$. Substituting Equations (10) and  (13) into Equation (3), Equation (3) can then be expressed as a system of binary quadratic equations with two unknowns $x_{11}\left(t_{n}\right)$ and $y(t_{n})$. Substituting the value from Equation (12) into Equation (3) to solve the system of equations yields

(14) $$\left\{\begin{array}{c} x_{11}\left(t_{n}\right)=\frac{d_{21}^{2}\left(t_{n}\right)-d_{11}^{2}\left(t_{n}\right)}{2d_{r}}-\frac{d_{r}}{2}\\ x_{21}\left(t_{n}\right)=\frac{d_{21}^{2}\left(t_{n}\right)-d_{11}^{2}\left(t_{n}\right)}{2d_{r}}+\frac{d_{r}}{2}\\ y\left(t_{n}\right)=\sqrt{d_{11}^{2}\left(t_{n}\right)-{\left(h_{t}-h_{r}\right)}^{2}-{\left(\frac{d_{21}^{2}\left(t_{n}\right)-d_{11}^{2}\left(t_{n}\right)}{2d_{r}}-\frac{d_{r}}{2}\right)}^{2}} \end{array}\right.$$

According to the positional relationship shown in Figure 3, the position of the shelf reference tag $T_{1}$ relative to the robot at time $t_{n}$ can be obtained as

(15) $${\hat{C}}_{1}\left(t_{n}\right)=\left[{\hat{x}}_{1}\left(t_{n}\right),{\hat{y}}_{1}\left(t_{n}\right),h_{t}\right]=\left[\frac{x_{11}\left(t_{n}\right)+x_{21}\left(t_{n}\right)}{2},y_{1}\left(t_{n}\right),h_{t}\right]$$

During the movement of the robot, the relative position between the antenna and the tag is constantly changing. When $d_{21}\left(t_{en}\right)=d_{11}\left(t_{en}\right)$, the calculation stops. The position coordinate $C_{1}\left(t_{n}\right)$ of tag $T_{1}$ in the coordinate system can be expressed as

(16) $$C_{1}\left(t_{n}\right)=\left[x_{1}\left(t_{n}\right),y_{1}\left(t_{n}\right),h_{t}\right]=\left[\frac{x_{11}\left(t_{n}\right)+x_{21}\left(t_{n}\right)}{2}+vt_{n},\frac{1}{n}\sum_{m=0}^{n}y\left(t_{m}\right),h_{t}\right],n\in \left[0,en\right]$$

According to the positional relationship, the position coordinate $C_{2}\left(t_{n}\right)$ of the tag $T_{2}$ can be obtained as

(17) $$C_{2}\left(t_{n}\right)=\left[x_{2}\left(t_{n}\right),y_{2}\left(t_{n}\right),h_{t}\right]=\left[\frac{x_{11}\left(t_{n}\right)+x_{21}\left(t_{n}\right)}{2}+vt_{n}+d_{t},\frac{1}{n}\sum_{m=0}^{n}y\left(t_{m}\right),h_{t}\right],n\in \left[0,en\right]$$

After obtaining the position coordinates of the shelf positioning tags $T_{1}$ and $T_{2}$, this paper regards the x-axis coordinate of the shelf positioning tag as the boundary of the shelf, thereby further identifying the behavior of the robot entering or leaving the shelf.

### 4.2. Identification of RFID Robot’s Shelf-Entering and Shelf-Leaving Behaviors

On the basis of obtaining the shelf position coordinates, to accurately identify the behavior of the RFID robot entering or leaving the shelf, the key lies in obtaining the two time nodes $t_{en}$ and $t_{out}$ when the robot enters or leaves the optimal reading area of the goods tags. Therefore, this section proposes a time estimation algorithm based on Multiple Linear Regression-Ordinary Least Squares (MLR-OLS) to estimate $t_{en}$ and $t_{out}$ by combining the position sensing model of robot-shelf interaction and the physical characteristics of RSSI.

#### 4.2.1. Zero Crossing Estimation Based on Linear Interpolation

Taking the robot entering the shelf as an example, according to the physical characteristics of the system, $d_{21}\left(t\right)>d_{11}\left(t\right)$ in the interval $t<t_{en}$, and $d_{21}\left(t\right)<d_{11}\left(t\right)$ in the interval $t>t_{en}$. Therefore, when the robot is at position $R(t_{en})$, have $d_{21}\left(t_{en}\right)=d_{11}\left(t_{en}\right)$. Based on this theory, the robot system continuously acquires $d_{11}$ and $d_{21}$ during its movement. As shown in Formula (18), by comparing $d_{11}$ and $d_{21}$ at the same moment, the moment $t_{en}$ when the robot enters the shelf can be obtained.

(18) $$\left\{\begin{array}{c} d_{11}=\left[d_{11}\left(t_{0}\right),\cdots ,d_{11}\left(t_{en}\right),\cdots ,d_{11}\left(t_{out}\right),\cdots ,d_{11}\left(t_{N}\right)\right]\\ d_{21}=\left[d_{21}\left(t_{0}\right),\cdots ,d_{21}\left(t_{en}\right),\cdots ,d_{21}\left(t_{out}\right),\cdots ,d_{21}\left(t_{N}\right)\right] \end{array}\right.$$

However, in actual scenarios, data is collected at discrete time points $t_{0},t_{2},\cdots ,t_{N}$, and there is inevitably measurement noise. It is almost impossible for $d_{11}$ and $d_{21}$ calculated by phase to be completely equal at any sampling point. Therefore, directly determining $t_{en}$ by searching for $d_{21}\left(t_{i}\right)=d_{11}\left(t_{i}\right)$ is unreliable. So, this paper adopts a zero-crossing estimation algorithm based on linear interpolation to obtain $t_{en}$, and the $t_{en}$ obtained by this method is denoted as the estimated value ${\hat{t}}_{en1}$. The steps are as follows:

Step 1: Construct the differential signal: First, define a new differential time sequence Δd(t). According to the signal characteristics, $t_{en}$ is the zero-crossing time when Δd(t) changes from a negative value to a positive value. Therefore, the original problem is transformed into solving the root of Δd(t)=0.

(19) $$\Delta d\left(t\right)=d_{11}\left(t\right)-d_{21}\left(t\right)$$

Step 2: Locate the interval: By traversing the differential dataset, find two consecutive sampling points where the sign of Δd(t) changes. That is, find the index *i* that satisfies the following conditions:

(20) $$\Delta d\left(t_{i}\right)<0,\;\mathrm{and}\;\Delta d\left(t_{i+1}\right)>0$$

From this, it can be determined that the time $t_{en}$ is within the time interval $\left(t_{i},t_{i+1}\right)$.

Step 3: Linear interpolation: It is assumed that within the sufficiently small sampling interval $\left(t_{i},t_{i+1}\right)$, the variation trend of the differential signal Δd(t) can be approximated as linear. Based on this assumption, the straight line formed by the two points $\left[t_{i},\Delta d\left(t_{i}\right)\right]$ and $\left[t_{i+1},\Delta d\left(t_{i+1}\right)\right]$ can be used to estimate the position of the zero-crossing, and the estimated value ${\hat{t}}_{en1}$ is calculated.

(21) $${\hat{t}}_{\mathrm{en}1}=t_{i}-\Delta d\left(t_{i}\right)\frac{t_{i+1}-t_{i}}{\Delta d\left(t_{i+1}\right)-\Delta d\left(t_{i}\right)}$$

Among them, $t_{i}$ and $t_{i+1}$ are the two time points before and after the located critical point ${\hat{t}}_{en1}$, and $\Delta d(t_{i})$ and $\Delta d(t_{i+1})$ are the corresponding differential signal values at these two time points. By performing linear interpolation between two discrete data points, this method can effectively overcome the small fluctuations caused by noise and the limitations brought by data discreteness.

#### 4.2.2. Estimation Based on Tag RSSI

Due to possible interference in the tag information collection process, there may be large errors in obtaining $t_{en}$ and $t_{out}$ using a single characteristic value. This section proposes to estimate $t_{en}$ and $t_{out}$ based on the RSSI of the tag.

UHF RFID communication is based on backscatter technology, and the RSSI of the tag is usually negatively correlated with the distance between the tag and the antenna. The longer the distance between the tag and the antenna, the smaller the RSSI value of the tag. This relationship can be derived through the Friis transmission equation and the free-space path loss model [30],

(22) $$\left\{\begin{array}{c} P_{r}=P_{t}G_{t}G_{r}{\left(\frac{\lambda }{4\pi d}\right)}^{2}\\ P=P_{0}-10n\log\left(\frac{d}{d_{0}}\right) \end{array}\right.$$

Among them, $P_{r}$ is the RSSI value returned by the tag received by the antenna, $P_{t}$ represents the signal power emitted by the reader, $G_{r}$ is the gain of the reader antenna, $G_{t}$ is the gain of the tag antenna, λ is the wavelength, *d* is the distance from the antenna to the tag, $P_{0}$ and $d_{0}$ are the RSSI value of the reference tag and its distance to the antenna, and *n* is the path loss exponent, usually between 2 and 4, depending on the environmental conditions.

**Figure 6:**
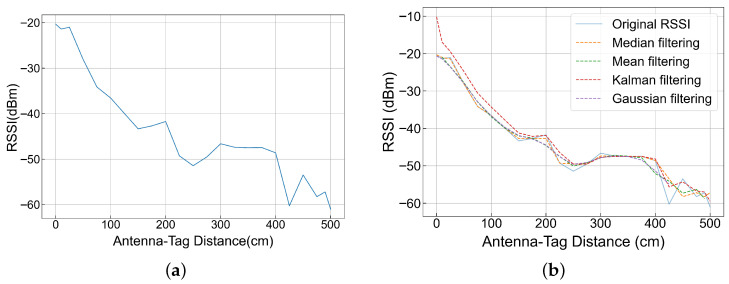
(**a**) Schematic diagram of the relationship between original RSSI and distance. (**b**) Schematic diagram of the relationship between RSSI filtering and distance.

In the actual communication of UHF RFID, the electromagnetic waves emitted by the reader will be reflected, refracted and other phenomena after encountering obstacles during propagation, resulting in the signal reaching the receiving ends of the tag and the reader through multiple paths. When the signal reaches the receiving end, it may be superimposed or canceled. In order to verify the above content, this paper simulates the warehouse environment for testing. The antenna is fixed, the distance between the tag and the antenna is moved from 0 cm to 500 cm, RSSI is collected every 10 cm, each measurement lasts for 30 s, and the average value of RSSI is taken after a total of 10 groups of data are tested. The results are shown in Figure 6a, which confirms that the RSSI does not show a strict linear relationship with the distance between the tag and the antenna in the actual environment.

In order to reduce the interference of the environment on RSSI, this paper processes the original RSSI. As shown in Figure 6b, it shows the results of removing outliers from the original RSSI through median filtering, mean filtering, Kalman filtering and Gaussian filtering, respectively. It can be seen from the figure that the RSSI has a better effect after Gaussian filtering. While retaining more details of the original RSSI, it makes the waveform smoother and presents a better linear relationship. Therefore, the RSSI values used in this paper are all values obtained after the original RSSI is processed by Gaussian filtering.

According to Formula (22), it is easy to get

(23) $$RSSI\propto \frac{1}{d^{n}}$$

**Figure 7:**
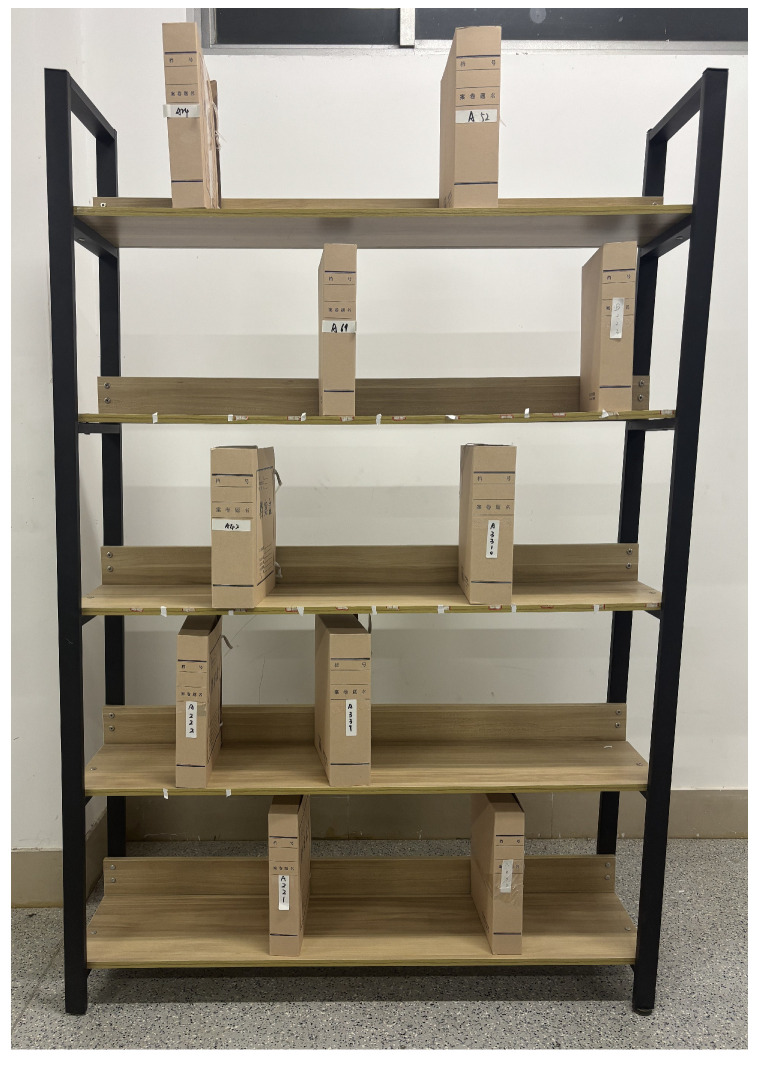
Multiple tags test scenario.

**Figure 8:**
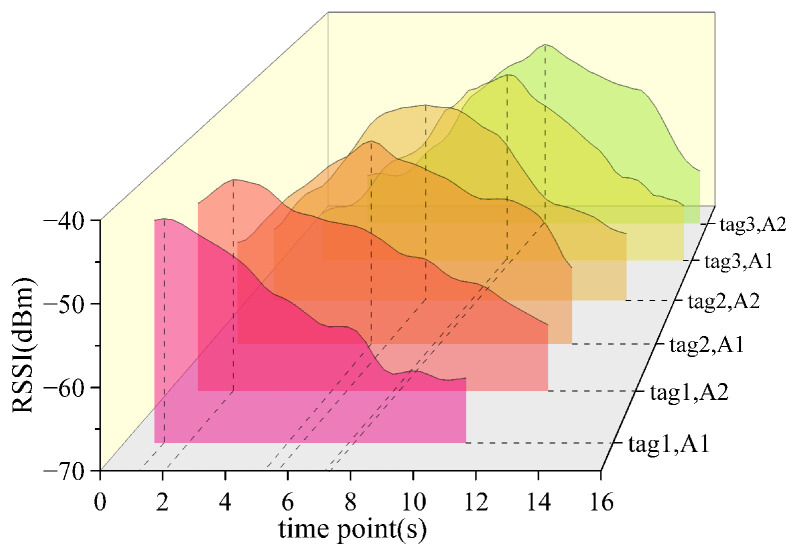
RSSI peak time corresponding to different tags.

This formula shows that RSSI has a logarithmic inverse relationship with distance d. The farther the distance between the tag and the antenna is, the smaller the RSSI will be, and the faster the RSSI attenuation speed will be. In order to further verify the universality of this formula in the warehouse scene with multiple tags, this paper simulates the warehouse environment for testing. As shown in Figure 7, multiple goods are randomly placed on the shelf, and each good is attached with an RFID tag. The robot is controlled to move from the right side of the shelf to the left side, and dual antennas are used to continuously collect the RSSI of each tag. This paper selects three tags with obvious differences in RSSI peaks. The test results are shown in Figure 8, which shows the RSSI change curves of the tags collected by antenna $A_{1}$ and antenna $A_{2}$.

It can be seen from Figure 8 that the RSSI peaks of tag1 obtained by antenna $A_{1}$ and antenna $A_{2}$ are approximately at 1.3 s and 2 s, the RSSI peaks of tag2 obtained by antenna $A_{1}$ and antenna $A_{2}$ are approximately at 5.2 s and 5.8 s, and the RSSI peaks of tag3 obtained by antenna $A_{1}$ and antenna $A_{2}$ are approximately at 7.1 s and 7.4 s. The average value of the times at which the two RSSI peaks of the tag occur is calculated to represent the time point when the robot is closest to the tag. That is, the time points when the robot is closest to tag1, tag2, and tag3 are 1.65 s, 5.5 s, and 7.25 s, respectively.

Using the above conclusions and methods, define the RSSI of tag $T_{j}$ collected by antenna $A_{i}$ at time $t_{n}$ as $RSSI_{ij}\left(t_{n}\right)$. All RSSI values collected by the RFID robot system during the entire operation process are represented as the following matrix:

(24) $$\begin{array}{c} RSSI_{ij}\left(t_{n}\right)=\left[RSSI_{ij}\left(t_{0}\right),\cdots ,RSSI_{ij}\left(t_{en}\right),\cdots RSSI_{ij}\left(t_{\mathrm{out}}\right),\cdots RSSI_{ij}\left(t_{N}\right)\right] \end{array}$$

Further,

(25) $$\left\{\begin{array}{c} RSSI_{1j}=\left[RSSI_{1j}\left(t_{0}^{\prime }\right),\cdots ,RSSI_{1j}\left(t_{\mathrm{en}}^{\prime }\right),\cdots RSSI_{1j}\left(t_{\mathrm{out}}^{\prime }\right),\cdots RSSI_{1j}\left(t_{N}^{\prime }\right)\right]\\ RSSI_{2j}=\left[RSSI_{2j}\left(t_{0}^{\prime \prime }\right),\cdots ,RSSI_{2j}\left(t_{\mathrm{en}}^{\prime \prime }\right),\cdots RSSI_{2j}\left(t_{\mathrm{out}}^{\prime \prime }\right),\cdots RSSI_{2j}\left(t_{N}^{\prime \prime }\right)\right] \end{array}\right.$$

Taking the robot entering the shelf as an example, in order to find the time $t_{en}$ when the robot enters the shelf, in Formula (25), select the two maximum values $MaxRSSI_{11}$ and $MaxRSSI_{21}$, then we can get

(26) $$\left\{\begin{array}{c} MaxRSSI_{11}=RSSI_{11}\left(t_{en}^{\prime }\right)\\ MaxRSSI_{21}=RSSI_{21}\left(t_{en}^{\prime \prime }\right) \end{array}\right.$$

Take the average of $t_{en}^{\prime }$ and $t_{en}^{\prime \prime }$, and the expression is as shown in Formula (27), then the time $t_{en}$ when the robot enters the shelf can be obtained. The $t_{en}$ obtained by this method is expressed as the estimated value ${\hat{t}}_{en2}$.

(27) $${\hat{t}}_{en2}=\frac{1}{2}\left(t_{en}^{\prime }+t_{en}^{\prime \prime }\right)$$

#### 4.2.3. Time Estimation Algorithm Based on MLR-OLS

After obtaining the times ${\hat{t}}_{en1}$ and ${\hat{t}}_{en2}$ when the robot enters the shelf through the above two methods, in order to further improve the estimation accuracy of $t_{en}$, this paper proposes a time estimation algorithm based on MLR-OLS. This algorithm can combine the estimated values $t_{en1}$ and $t_{en2}$ to obtain a more accurate estimated value ${\hat{t}}_{en}$.

Multiple Linear Regression (MLR) is a classic statistical modeling method, which aims to predict the dependent variable through the linear combination of multiple independent variables. Its core assumption is that there is a linear relationship between the dependent variable and the independent variables, and the observation noise follows a normal distribution with a mean value of 0. In this paper, the goal is to use the estimated value ${\hat{t}}_{en1}$ and the estimated value ${\hat{t}}_{en2}$ to obtain the accurate time $t_{en}$ when the robot enters the shelf, and the following regression model is established:

(28) $${\hat{t}}_{en}=\beta_{0}+\beta_{1}{\hat{t}}_{en1}+\beta_{2}{\hat{t}}_{en2}+\varepsilon$$

In the formula, ${\hat{t}}_{en}$ is the fusion estimation time of the model, that is, the dependent variable; ${\hat{t}}_{en1}$ and ${\hat{t}}_{en2}$ are the two input features of the model, which, respectively, represent the preliminary estimated values obtained by the aforementioned two methods, that is, the independent variables; $\beta_{0}$ is the intercept term, representing the inherent deviation of the system; $\beta_{1}$ and $\beta_{2}$ are regression coefficients, reflecting the contribution weights of each independent variable to $t_{en}$; ε is the residual term, representing the part of the variation that cannot be explained by the model.

The model weights are obtained by Ordinary Least Squares (OLS). OLS is the most basic linear regression method. Its goal is to find the regression model parameters, so as to minimize the Residual Sum of Squares between the model predicted values and the true values. The formula is

(29) $$\min_{\beta_{0},\beta_{1},\beta_{2}}\sum_{i=1}^{N}{\left(t_{\mathrm{en}}^{\left(i\right)}-\left(\beta_{0}+\beta_{1}t_{\mathrm{en}1}^{\left(i\right)}+\beta_{2}t_{\mathrm{en}2}^{\left(i\right)}\right)\right)}^{2}$$

In the formula, *N* is the number of samples. Calculate the optimal parameter $\beta ={\left[\beta_{0},\beta_{1},\beta_{2}\right]}^{T}$, and its closed-form solution is

(30) $$\hat{\beta }={\left(X^{T}X\right)}^{-1}X^{T}y$$

In the formula, the matrix $X\in R^{N\times 3}$ contains the intercept term and the normalized eigenvalues; that is, each row is $\left[1,t_{en1}^{\left(i\right)},t_{en2}^{\left(i\right)}\right]$, $\hat{\beta }={\left[{\hat{\beta }}_{0},{\hat{\beta }}_{1},{\hat{\beta }}_{2}\right]}^{T}$ is the parameter estimation value, and $y={\left[t^{\left(1\right)},t^{\left(2\right)},\cdots ,t^{\left(N\right)}\right]}^{T}$ is the observation vector.

The time estimation algorithm based on MLR-OLS obtains the estimated value ${\hat{t}}_{en}$ of the time point when the RFID robot enters the shelf, and indirectly realizes the recognition of the RFID robot’s behavior of entering the shelf. Similarly, using the above method, when the RFID robot leaves the shelf, the recognition of the RFID robot’s behavior of leaving the shelf can also be realized.

## 5. Distribution Sensing Model of Goods Tags

When the robot moves to the center of the optimal reading area for goods tags, it stops moving and starts perceiving the distribution of goods on the shelves. The density of tag groups has a significant impact on the performance of the RFID system; this section proposes a distribution sensing model of goods tags. The model takes the RSSI of successfully read goods tags and the number of reads per unit time as sensing features, uses a density clustering algorithm to perceive the goods distribution in each area on the shelves, divides the shelf area into goods missing read risk areas of different levels, estimates the number of missing goods using time slots, and finally designs an adaptive reading strategy for the RFID robot.

### 5.1. Experimental Observation

#### 5.1.1. Experimental Test

**Figure 9:**
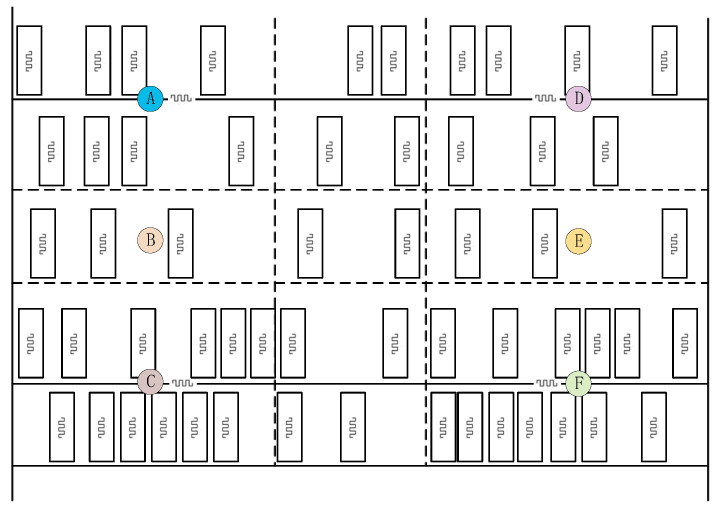
Schematic diagram of test scenario.

**Figure 10:**
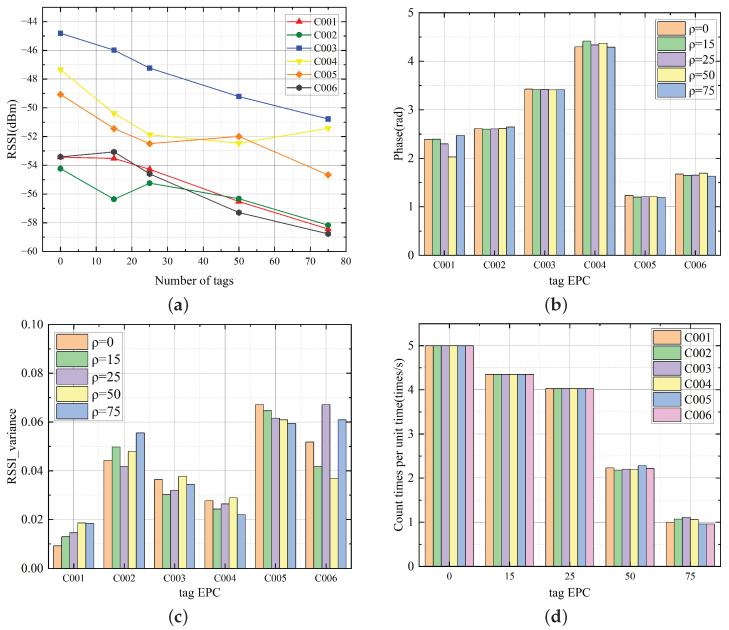
Graph of eigenvalue change results of shelf reference tags under different densities: (**a**) Graph of RSSI change results of shelf reference tags. (**b**) Graph of phase change results of shelf reference tags. (**c**) Graph of RSSI variance change results of shelf reference tags. (**d**) Graph of read count change results of shelf reference tags.

To study the influence of the law of tag density on the RFID system, this paper conducts multiple inventory tests for five different tag densities and collects the characteristic data of shelf reference tags in different areas. The test scenario is shown in Figure 9. The test environment is set as an open space with a shelf placed in it. The shelf is divided into six sub-areas, and a shelf reference tag is pasted in the center of each sub-area. Goods are randomly distributed in each area of the shelf, and each good is pasted with a goods tags. The test results are shown in Figure 10, and the experimental results show the following:

(1) There is significant spatial heterogeneity in the RSSI of shelf reference tags in different areas. The tag density ρ has a significant negative correlation with RSSI. As the tag density ρ increases, the RSSI shows a decreasing trend.
(2) There is significant spatial heterogeneity in the phase of shelf reference tags in different areas. However, under different density conditions, the phase is relatively stable and has low sensitivity to density.
(3) The RSSI variance of shelf reference tags in different areas is slightly different, but the RSSI variance of all tags is lower than 0.07 under different densities, and there is no obvious correlation between the RSSI variance and the tag density ρ.
(4) The tag density ρ has a significant negative correlation with the number of times tags are read per unit time. The number of reads shows a decreasing trend as the tag density ρ increases. When ρ≤25, the number of reads is above 4 times and relatively stable. When ρ≥50, the attenuation rate of the number of reads accelerates as the density increases.

Based on the analysis results of the aforementioned multi-dimensional data, this paper takes the RSSI and the number of times tags are read per unit time as the sensing features of tag density.

#### 5.1.2. Experimental Theoretical Analysis

In order to further explain the phenomena observed in the experiment, this paper makes a theoretical analysis from the working mechanism of RFID system as follows:

(1) Diminishing Marginal Energy Effect: In the RFID system, the energy coverage of the reader antenna is divided into the main lobe energy area and the side lobe energy area. The beam emitted by the antenna is mapped to the tag plane as a circular area, and its energy intensity decreases gradiently from the core area to the periphery. When the tag is located in the main lobe energy area, it can obtain sufficient and stable excitation power. However, when the tag is located in the side lobe energy area, if the energy received by the tag is insufficient to support its continuous and stable communication, the RSSI value will decrease, and even the situation of unrecognizability will occur.
(2) Backscatter Energy Competition: The RFID system realizes tag identification based on the backscatter communication principle. When the number of tags is small, energy can be effectively concentrated on the tags to stimulate their stable backscatter response. However, when the tag density increases, the excitation energy that each tag can obtain does not increase. Instead, it will decrease due to the average distribution of total energy, resulting in a decrease in the tag activation probability.
(3) Tag Response Time Slot Collision: In the RFID system, the reader usually uses the anti-collision mechanism of the frame-based slotted ALOHA protocol to poll and read tags. This protocol relies on tags randomly selecting one to respond in the allocated time slots. However, when the tag density ρ increases, the limited time slot resources will face higher concurrent response pressure, causing the response signals of multiple tags to collide in the same time slot, so that the reader cannot successfully identify any tag. The increase in the frequency of such time slot collisions will directly reduce the overall identification efficiency of the RFID system, which in turn manifests as a decrease in the number of times tags are read per unit time.

### 5.2. Goods Tags Density Classification Method Based on K-Means

Based on the above analysis, in order to realize the sensing of goods distribution, this paper proposes a goods tag density classification method based on the K-means. This method perceives the goods distribution in each area on the shelf based on the eigenvalues of the shelf reference tags. The method flow is shown in Algorithm  1.

In this algorithm, when the number of reads and RSSI corresponding to a certain cluster center are significantly higher than those of other categories, this category can be determined as a high-density area, followed by a medium-density area, and finally a low-density area, which are labeled as class I, class II, and class III, respectively. Then, the classification results are analyzed to divide the shelf area into regions of different density levels. This paper focuses on the missing read risk in high-density areas. Theoretically, when the tag density is high, missing tag reads will occur. Therefore, the high-density area of goods tags is marked as a high-risk area for missing reads.

### 5.3. Estimation of the Number of Missing Read Tags

For the high-risk area, this section proposes a missing tag count estimation algorithm based on frame states and random forest by leveraging the time slot state distribution characteristics of the frame-based slotted ALOHA protocol in the MAC layer.
**Algorithm 1** Goods Tags Density Classification Method Based on K-Means**Require:** Reference tag feature dataset $X=\{x_{1},x_{2},\ldots ,x_{n}\}$, where $x_{i}=\left\{rssi_{i},freq_{i}\right\}$; set number of clusters K=3
**Ensure:** Density category for each reference tag: class I, class II, class III
  1:Initialization: Randomly select 3 samples from the dataset as initial cluster centers $\left\{\mu_{1},\mu_{2},\mu_{3}\right\}$  2:**repeat**  3:    **for** Each reference tag $x_{i}$ **do**  4:       Calculate its Euclidean distance to all cluster centers:$$d_{j}=∥x_{i}-\mu_{j}∥,\;j=1,2,3$$  5:       Assign $x_{i}$ to the cluster $C_{k}$ with the smallest distance, where $k=\arg\min_{j}d_{j}$  6:    **end for**  7:    **for** Each cluster $C_{k}$ **do**  8:       Update its cluster center:$$\mu_{k}^{\prime }=\frac{1}{|C_{k}|}\sum_{x_{i}\in C_{k}}x_{i}$$  9:       **if** $\mu_{k}^{\prime }=\mu_{k}$ **then**10:           Update current cluster center $\mu_{k}$ to $\mu_{k}^{\prime }$11:       **else**12:           Keep current cluster center unchanged13:       **end if**14:    **end for**15:**until** Cluster centers no longer change or maximum iterations reached16:**return** Density classification for each reference tag (class I/class II/class III)


#### 5.3.1. Statistical Analysis of Frame States

In the RFID inventory based on the Frame Slotted ALOHA (FSA) protocol, the reader transmits an inquiry frame containing *L* time slots, which is regarded as starting a round of inventory. During the inventory process, the time slots of this frame can be divided into three states: idle time slot (a time slot with no tag response), successful time slot (a time slot with exactly one tag response), and collision time slot (a time slot with signal collision caused by responses of two or more tags). Assuming the total number of tags is *N*, each tag responds independently when being inventoried, and independently selects a time slot to respond with an equal probability of $\frac{1}{L}$, then the probability that exactly *k* tags respond in the *i*-th time slot follows a binomial distribution $K_{i}∼B\left(N,\frac{1}{L}\right)$.

(31) $$P\left(K_{i}=k\right)=C_{N}^{k}{\left(\frac{1}{L}\right)}^{k}{\left(1-\frac{1}{L}\right)}^{N-k}$$

When both *N* and *L* are large, the above binomial distribution can be approximated as a Poisson distribution with an arrival rate $\lambda =\frac{N}{L}$, that is, $K_{i}∼P\left(\frac{N}{L}\right)$,

(32) $$P\left(K_{i}=k\right)\approx \frac{\lambda^{k}}{k!}e^{-\lambda }$$

Then, in one inventory frame, the probabilities that any time slot becomes an idle time slot, a successful time slot, and a collision time slot, $P_{e}$, $P_{s}$, and $P_{c}$, respectively, are

(33) $$P_{e}=P\left(K_{i}=0\right)=C_{N}^{0}{\left(\frac{1}{L}\right)}^{0}{\left(1-\frac{1}{L}\right)}^{N}={\left(1-\frac{1}{L}\right)}^{N}$$

(34) $$P_{s}=P\left(K_{i}=1\right)=C_{N}^{1}{\left(\frac{1}{L}\right)}^{1}{\left(1-\frac{1}{L}\right)}^{N-1}=\frac{N}{L}{\left(1-\frac{1}{L}\right)}^{N-1}$$

(35) $$P_{c}=1-P_{s}-P_{e}=1-\frac{N}{L}{\left(1-\frac{1}{L}\right)}^{N-1}-{\left(1-\frac{1}{L}\right)}^{N}$$

When both *N* and *L* are large, the probabilities that any time slot becomes an idle time slot, a successful time slot, and a collision time slot, $P_{e}$, $P_{s}$, and $P_{c}$, respectively, are

(36) $$P_{e}=P\left(K_{i}=0\right)=e^{-\lambda }$$

(37) $$P_{s}=P\left(K_{i}=1\right)=\lambda e^{-\lambda }$$

(38) $$P_{c}=1-P_{s}-P_{e}=1-e^{-\lambda }-\lambda e^{-\lambda }$$

Therefore, after one inventory frame ends, the expected values $E_{e}$, $E_{s}$, and $E_{c}$ of idle time slots, successful time slots, and collision time slots satisfy $L=E_{e}+E_{s}+E_{c}$, and they are, respectively,

(39) $$E_{e}=L\times P_{e}=L{\left(1-\frac{1}{L}\right)}^{N}$$

(40) $$E_{s}=L\times P_{s}=N{\left(1-\frac{1}{L}\right)}^{N-1}$$

(41) $$E_{c}=L\times P_{c}=L-L{\left(1-\frac{1}{L}\right)}^{N}-N{\left(1-\frac{1}{L}\right)}^{N-1}$$

When both *N* and *L* are large, the expected values $E_{e}$, $E_{s}$, and $E_{c}$ of idle time slots, successful time slots, and collision time slots are, respectively,

(42) $$E_{e}=L\times P_{e}=Le^{-\lambda }$$

(43) $$E_{s}=L\times P_{s}=L\lambda e^{-\lambda }$$

(44) $$E_{c}=L\times P_{c}=L\left(1-e^{-\lambda }-\lambda e^{-\lambda }\right)$$

The traditional method for tag number estimation usually observes and counts the distribution states of three types of time slots (idle slots, successful slots, and collision slots) within a frame, and infers the total number of tags based on their mathematical expectations under the ideal model. However, the performance of such methods relying on theoretical models will be affected by interference factors in practical applications.

#### 5.3.2. Algorithm for Estimating the Number of Missing Read Tags

To get rid of the strict dependence on the ideal ALOHA model, this paper analyzes the command interaction process between the reader and tags, counts the frame length of each frame in the MAC layer of goods tags per unit time, the number of time slots in each frame, and the time slot types (successful time slots, collision time slots, and idle time slots), and combines random forest to realize the estimation of the number of missing read tags.

Random forest is an ensemble learning method that performs classification or regression prediction by constructing and combining multiple random decision trees. It introduces “randomness” to reduce the overfitting risk of a single decision tree, thereby improving the accuracy and generalization ability of the model. Its core idea includes two key randomization processes: Bootstrap Aggregating and Random Feature Selection. The algorithm flow is as follows:

Step 1: Assume that the original dataset contains *M* samples. The Bootstrap sampling method is used for random sampling with replacement to generate multiple training sets. Bootstrap sampling randomly selects *D* samples from the original dataset to form a new training set $D_{train}={\left\{\left(x_{i},N_{i}\right)\right\}}_{i=1}^{M}$, where $x_{i}$ is the feature vector of the *i*-th area, and $N_{i}$ is the actual total number of tags.

Step 2: When constructing each decision tree, the random forest randomly selects a part of features during the splitting process of each node to form the input feature vector $x=[FL,E_{e},E_{s},E_{c},P_{e},P_{s},P_{c}]$, where FL, $E_{e}$, $E_{s}$, $E_{c}$, $P_{e}$, $P_{s}$, and $P_{c}$ are the frame length, the number of idle time slots, successful time slots, collision time slots, and their respective proportions.

Step 3: Initialize the random forest model set RF←⌀, and set the hyperparameters of the random forest: the number of decision trees *B*, the number of features *m* considered in each split, etc.

Step 4: For each decision tree $TR_{b}\left(b=1,2,\cdots ,B\right)$, perform Bootstrap sampling, sample with replacement from the original training set $D_{train}$ to construct a training subset $D_{b}$, while keeping the total number of samples unchanged. In this way, *B* different training sets $\left[D_{b}^{\left(1\right)},D_{b}^{\left(2\right)},\cdots ,D_{b}^{\left(B\right)}\right]$ can be generated, and each training set is used to train a decision tree.

Step 5: Use $D_{b}$ and $x_{i}$ to train a regression decision tree $TR_{b}$. When splitting each node, randomly select *m* candidate features from all features, and select the best splitting feature and splitting threshold according to the Mean Squared Error (MSE) criterion.

Step 6: Continue to grow the decision tree until the stopping condition is met.

Step 7: Add the trained sub-tree $T_{b}$ to the random forest model set. Finally, the set composed of *B* trained decision trees $RF=\{TR_{1},TR_{2},\cdots ,TR_{B}\}$ is the trained random forest model.

Step 8: Extract the feature vector $x_{new}=\left[FL,E_{e},E_{s},E_{c},P_{e},P_{s},P_{c}\right]$ from the obtained tag feature information and input it into the trained random forest to obtain the output of each decision tree,

(45) $${\hat{N}}_{b}=T_{b}\left(x\right),\;\left(b=1,2,\cdots ,B\right)$$

Step 9: Calculate the average output of all decision trees as the estimated total number of tags,

(46) $${\hat{N}}_{RF}=\frac{1}{B}\sum_{b=1}^{B}{\hat{N}}_{b},\;\left(b=1,2,\cdots ,B\right)$$

Step 10: Finally, use the estimated total number of tags to calculate the number of missing read tags,

(47) $${\hat{N}}_{\mathrm{miss}}={\hat{N}}_{\mathrm{RF}}-N_{\mathrm{read}}$$

### 5.4. RFID Robot Adaptive Reading

The preceding chapters successfully realize tag density classification and missing goods calculation in shelf areas through physical layer and MAC layer features. This section feeds back the sensing results to the RFID robot control system to perform targeted reading of goods in high-risk areas, thereby reducing the overall missing read rate of goods without significantly increasing the total inventory time.

After the mobile robot completes the first round of inventory and goods tag state sensing, it converts the different missing read risk levels divided in Section 5.2 into dwell time $V_{i}$, which is used as the decision weight for RFID robot path planning, as shown in Table 3.

This paper sets two preset inventory points $L_{k}$ to perform targeted reading of goods in missing read risk areas. Point $L_{1}$ is responsible for the area, $Area\left(L_{1}\right)=\left\{A_{1},A_{2},A_{3}\right\}$, and point $L_{2}$ is responsible for the area $Area\left(L_{2}\right)=\left\{A_{4},A_{5},A_{6}\right\}$. The dwell time $VL_{k}$ of each point is determined by the highest risk level in the area it contains.

(48) $$\;VL_{k}=\max_{i\in Area(L_{k})}\left(V_{i}\right)\;$$

According to the dwell decision value of point $L_{k}$, assign a dwell time $T_{stay,k}$ to each point as follows:

(49) $$T_{stay,k}=\left\{\begin{array}{cc} T_{H}, & VL_{k}=V_{H}\\ T_{M}, & VL_{k}=V_{M}\\ 0, & VL_{k}=V_{L} \end{array}\right.$$

Among them, $T_{H}>T_{M}>0$. The inventory priority of points is determined by the robot’s movement direction. The robot in this paper moves from left to right, so the inventory priority is $L_{2}>L_{1}$. During the access process, the robot will perform different inventory actions according to the dwell time of the points:

(1) If $T_{stay,k}>0$, that is, the point contains medium and high-risk areas, the robot will perform the action of approaching the shelf and stay for $T_{stay,k}$ duration to perform inventory.

(2) If $T_{stay,k}=0$, that is, all areas contained in the point are low-risk, the robot will pass quickly without staying to save inventory time.

## 6. Experimental Results and Analysis

### 6.1. Experimental Scenario Setup

**Figure 11:**
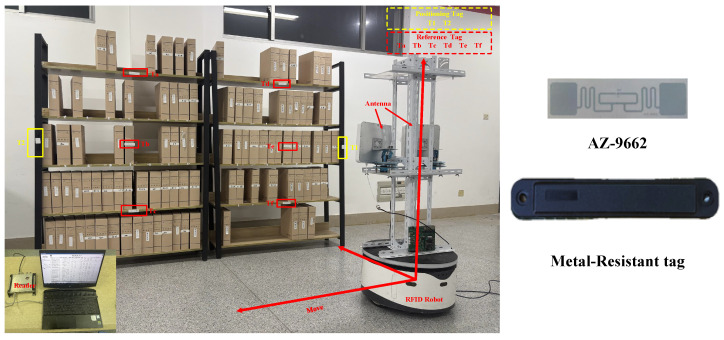
Experimental equipment and scenario diagram.

**Figure 12:**
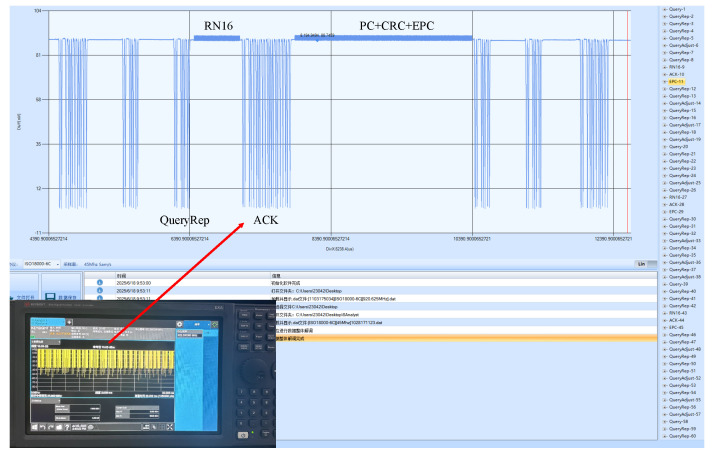
Schematic diagram of demodulating time slot information with spectrum analyzer.

To restore a real warehousing scenario, this paper builds the experimental scenario indoors. The experimental equipment and scenario diagram are shown in Figure 11. Two shelves are placed side by side indoors. Each shelf has five layers, with a length of 1.25 m, a height of 2 m, and a layer height of 0.4 m. Fifty goods with passive RFID tags attached to the sides are randomly placed on the shelves. One anti-metal tag is pasted on each side of the shelf, and one passive RFID tag is pasted in each of the six areas on the shelf. The models of experimental equipment are shown in Table 4. The robot is equipped with two antennas and moves from the left front of the shelf to the right at a uniform speed. During the whole process, the antennas continuously collect tag information. The experimental parameter settings are shown in Table 5. At the same time, a spectrum analyzer is used to capture the interaction process between the reader and the tags, and the interaction signals are demodulated to analyze the time slot information, as shown in Figure 12.

### 6.2. Model Verification

Based on the above experimental scenarios and parameter configurations, this section verifies the effectiveness of the position sensing model of robot-shelf interaction and the distribution sensing model of goods tags proposed in this paper through a series of experiments.

#### 6.2.1. Verification of the Position Sensing Model of Robot-Shelf Interaction

**Figure 13:**
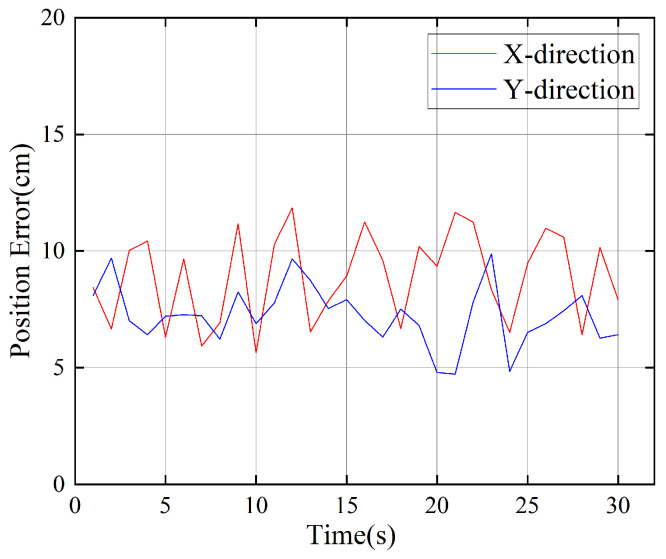
Graph of shelf position error.

This paper uses shelf positioning tags to locate the shelf and conducts 30 repeated experiments. The position errors in the X-axis and Y-axis directions are shown in Figure 13. It can be seen from the figure that the error range in the X direction is mainly distributed between 5 cm and 12 cm, with an average error of 8.7 cm. The errors in the Y direction are all less than 10 cm, with an average error of 7.2 cm. This error range is sufficient to meet the system’s reliable identification of the robot’s entry and exit behavior from the shelf.

**Figure 14:**
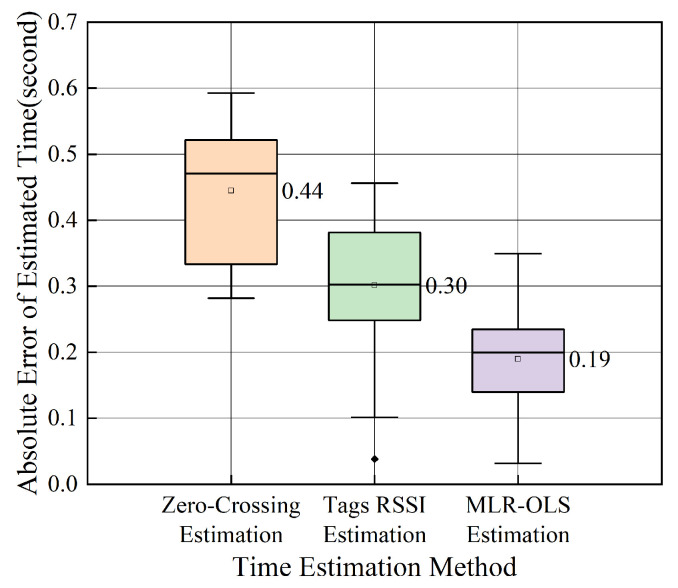
Graph of error comparison of different time estimation methods.

For the behavior recognition of RFID robots entering and exiting the shelf, this paper compares three different time estimation methods: zero-crossing estimation based on linear interpolation, estimation based on tag RSSI, and time estimation based on MLR-OLS. Figure 14 shows the average absolute error between the estimated time and the real time of the three methods in 30 groups of experiments. It can be observed from the figure that the estimation method with a single eigenvalue has large errors and instability, while the time estimation algorithm based on MLR-OLS further proposed in this paper based on two estimation methods has an average error of only 0.19 s, and the error distribution is more concentrated. The small time error can ensure that the robot accurately reaches the center position of the best reading area of the goods tags, providing a guarantee for the data collection of the distribution sensing model of goods tags.

#### 6.2.2. Verification of the Distribution Sensing Model of Goods Tags

**Figure 15:**
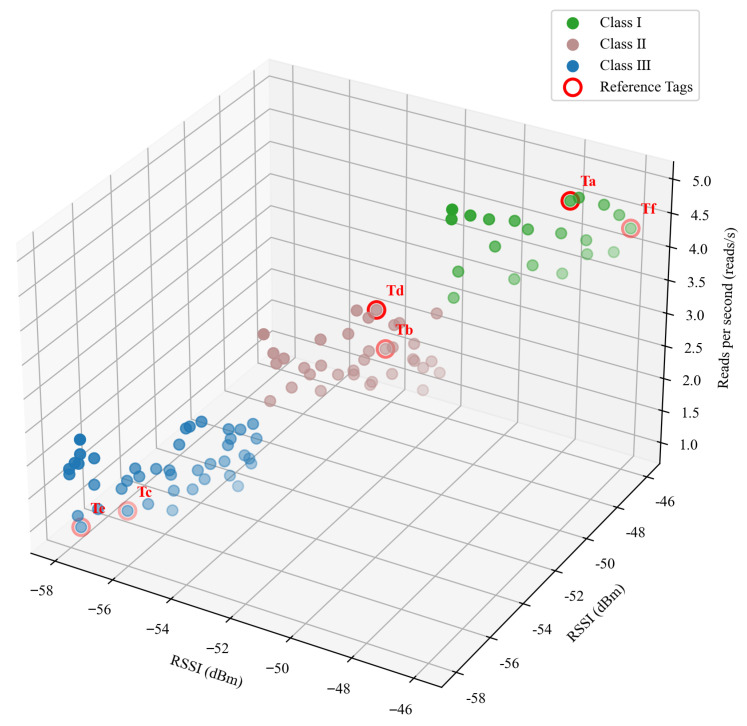
Results of goods tags density classification.

This paper extracts the average RSSI and the number of reads per second of goods tags and shelf reference tags (tag EPC: C001-C006), and analyzes them using the goods tags density classification method based on the K-means proposed in this paper. The experimental results are shown in Figure 15. It can be observed from the figure that the goods tags in the feature space are aggregated into three clusters: Class I is characterized by high RSSI and high reading rate, indicating that the tags in this cluster are in low-density areas; Class III is characterized by low RSSI and low reading rate, indicating that the tags in this cluster are in high-density areas. This verifies that the features selected in this paper can effectively distinguish different tag densities.

Based on this experiment, the mapping relationship between density ranges and tag missing read risk levels is established, as shown in Table 6. This mapping relationship is set according to the phenomenon that higher tag density leads to more intense channel competition, thereby resulting in a higher missing read probability.

**Figure 16:**
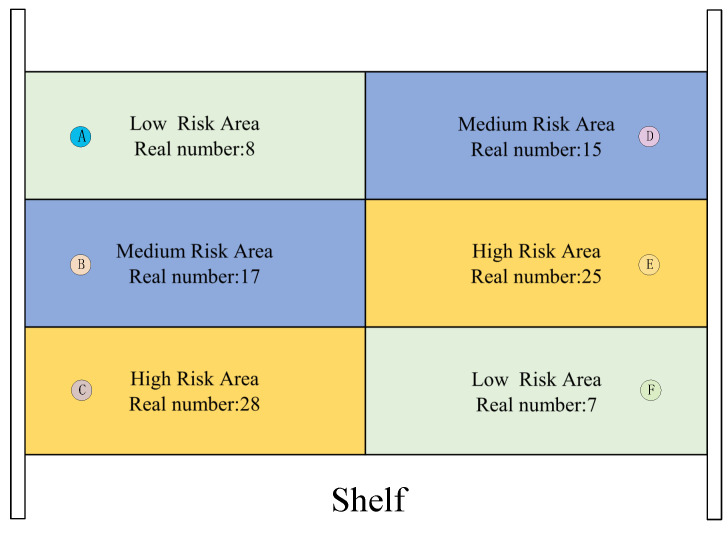
Results of missing read risk level assessment.

Figure 16 shows a missing read risk level assessment map in a typical experiment, where the shelf reference tags (marked with red circles in Figure 15) are classified into different tag density categories, and the clustering results are mapped back to the shelf area. Each cell in the figure represents a sub-area of the shelf, which contains the missing read risk level determined by the system, and the actual number of tags in each area is labeled for verification.

To verify the algorithm for estimating the number of missing read tags based on frame states and random forests proposed in this paper, this section conducts a series of experiments. The experimental settings cover different numbers of tags (5, 10, 25, 40, 55, 70, 85, 100, 115, 130, 145, 160) and multiple time slot frame lengths (16, 32, 64, 128). In each group of experiments, the time slot information within one frame is collected, including idle time slots, successful time slots, and collision time slots. Each experimental setting is repeated 30 times, and a total of 1440 sets of data are collected.

**Figure 17:**
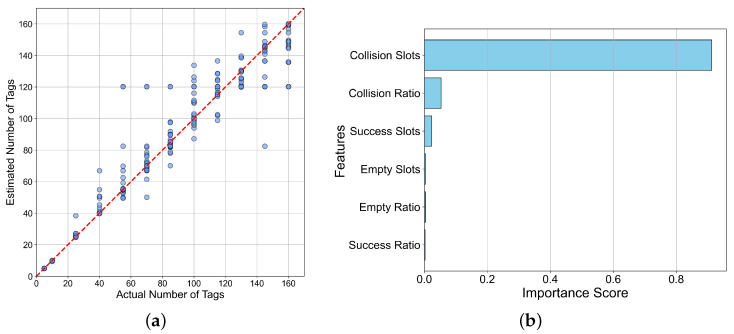
Performance and interpretability analysis of the proposed random forest model for estimating the number of missing read tags: (**a**) Graph of model estimation performance results. (**b**) Analysis of feature contribution.

The data is input into the constructed estimation algorithm, and the estimation performance is shown in Figure 17a. The determination (R^2^) of the model reaches 0.93 and an average estimation error of 6.82 for the number of tags. Figure 17b shows the contribution of multiple input time slot features in the algorithm to the estimation results. The results show that the contribution of the “collision time slot” feature to the estimation results exceeds 92%, which is the main factor affecting tag missing reads. This result conforms to the basic principle of the slotted ALOHA protocol in RFID communication, further verifying the credibility and practicality of the algorithm.

**Figure 18:**
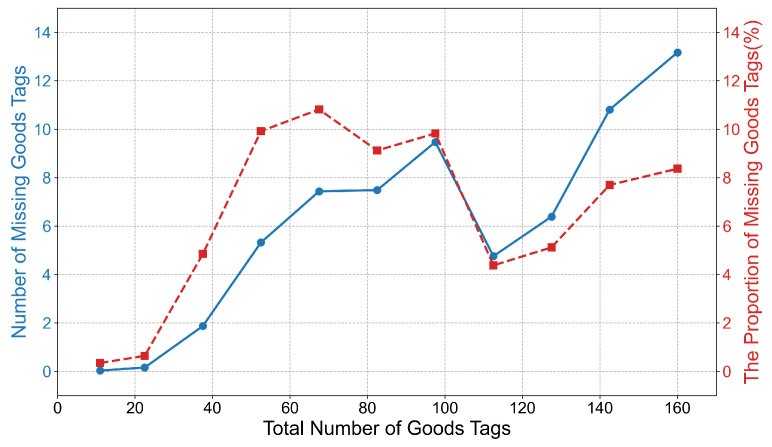
Estimation results of the algorithm under different numbers of tags.

To comprehensively evaluate the stability and reliability of the proposed estimation algorithm in various complex scenarios, this paper conducts experiments under different tag density conditions to simulate scenarios with goods from sparse to dense. As shown in Figure 18, the experimental results indicate that as the number of tags increases, the number of missing read tags also increases. However, when the number of tags increases to approximately 95, the proportion of missing read tags decreases instead, and the proportion of missing read tags remains at a relatively stable level in the experiment. This paper infers that this phenomenon may be due to the dynamic Q algorithm built into the commercial reader used.

**Figure 19:**
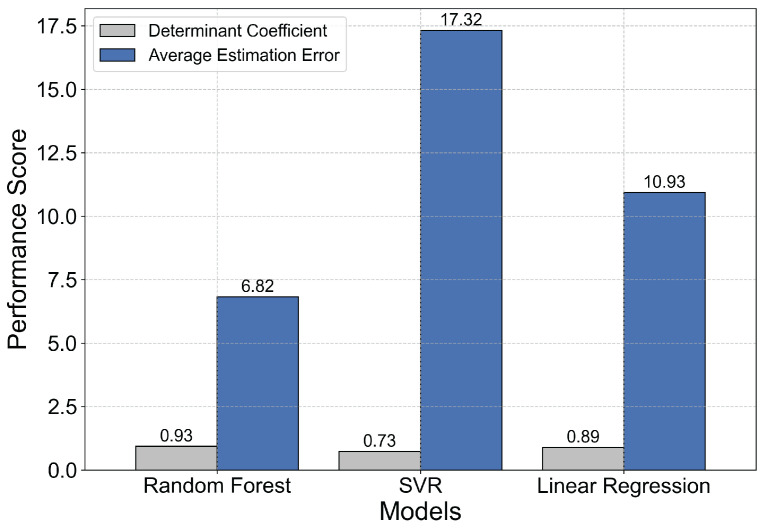
Performance comparison of different models.

In addition, to verify the rationality of the model selected in the algorithm, this paper selects Support Vector Regression (SVR) and Multiple Linear Regression as comparison models to evaluate the performance of the three models. The results are shown in Figure 19. The experimental results show that the random forest model used in this paper is superior to the SVR and Linear Regression models in two key indexes, which verifies its applicability as a solution to the problem studied in this paper.

**Figure 20:**
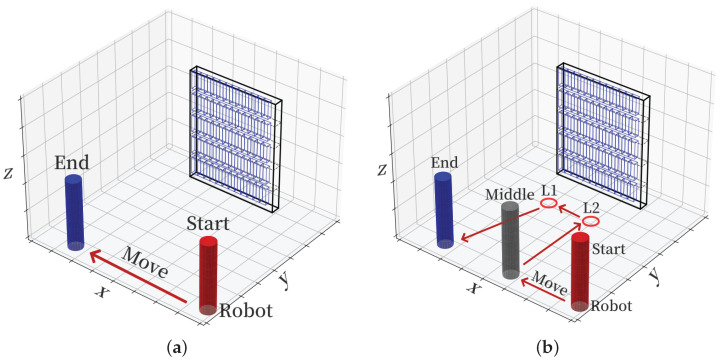
Comparison diagram of robot path planning: (**a**) Original path. (**b**) Optimized path.

To objectively evaluate the performance gain of the proposed method, for the RFID adaptive inventory strategy, this paper sets up a normal inventory mode and an adaptive inventory mode for comparison, as shown in Figure 20. In Figure 20a, the robot moves from the left to the right at a fixed speed without any stay or position adjustment. In Figure 20b, the robot accesses points $L_{2}$ and $L_{1}$ in sequence and stays for different inventory times $T_{stay,k}$ at each point. This paper implements the adaptive inventory strategy based on the missing read risk level evaluation results in Figure 16, where point $L_{1}$ contains the high missing read direction area *C* and point $L_{2}$ contains the high missing read direction area *E*. The robot accesses $L_{2}$ and $L_{1}$ in sequence and stays for inventory at each point. Experimental results show that the final missing read rate of the adaptive inventory mode is always lower than that of the normal inventory mode, especially in scenarios where tags are concentrated in high-risk areas; the missing read rate of the adaptive mode is significantly lower than that of the normal mode.

## 7. Conclusions

Aiming at the challenges faced by mobile RFID robots in shelf inventory tasks in warehouse environments, this paper proposes Physical–MAC layer integration: a cross-layer sensing method for mobile UHF RFID robot reading states based on MLR-OLS and random forest. This method includes two core models: a position sensing model of robot-shelf interaction and a distribution sensing model of goods tags. In the position sensing model of robot-shelf interaction, a spatial rectangular coordinate system is established, a time estimation algorithm based on MLR-OLS is designed, and the phase features and RSSI of shelf positioning tags are used to estimate the position coordinates of the shelf and identify the behavior of the RFID robot entering and exiting the shelf. In the distribution sensing model of goods tags, the feature information of the physical layer and MAC layer of tags is utilized, a K-mean-based goods tag density classification method is proposed to obtain the distribution of goods tags and evaluate the goods missing read risk levels of shelf sub-areas, and a missing tag count estimation algorithm based on frame states and random forest is designed to estimate the number of missing goods tags. Finally, an adaptive reading strategy for RFID robots is proposed, which converts the tag density risk levels into decision values of dwell time for optimized points to perform targeted reading of missing goods. Based on the above work, this paper realizes cross-layer state sensing and reading optimization of the mobile RFID robot system. Future research will further explore adaptive inventory strategies suitable for dynamically changing environments to improve the reading accuracy and inventory efficiency of RFID robot systems in application scenarios such as intelligent retail and smart warehousing.

## Figures and Tables

**Table 1:** Tag definition.

Fixed Tags	Name
Shelf Reference Tags	$T_{a}$, $T_{b}$, $T_{c}$, $T_{d}$, $T_{e}$, $T_{f}$
Shelf Positioning Tags	$T_{1}$, $T_{2}$

**Table 2:** Coordinate axis direction description.

Axis	Description
X-axis	Initial traveling direction of the robot
Y-axis	Direction of antenna reading tag
Z-axis	Vertical and upward direction perpendicular to the ground

**Table 3:** Path planning residence time.

Missing Read Risk Level	Dwell Time Decision Value
High Risk	$V_{H}$
Medium Risk	$V_{M}$
Low Risk	$V_{L}$

**Table 4:** Experimental equipment models.

Device	Parameter
Shelf Reference Tag	AZ-9662
Shelf Positioning Tag	ES-ABS13522 UHF Metal-Resistant tag
goods Tags	AZ-9662
Reader	Impinj SpeedWay R420
Antenna	9 dBi Circularly Polarized Antenna
Mobile Robot	Water Robot
spectrum analyzer	Keysight N9010B
Protocol	ISO-18000-6C

**Table 5:** Experimental parameter settings.

Parameter	Value	Unit
Reader Read Power	26	dBm
Reader Frequency	920.625	MHz
Robot Speed	0.2	m/s
Antenna Height	1.1	m
Shelf Positioning Tag Height	1	m

**Table 6:** Density classification.

Category	Density Range	Missed Detection Risk Level
Class I	ρ<10	Low Risk
Class II	10≤ρ≤20	Medium Risk
Class III	ρ>20	High Risk

## Data Availability

The data supporting the reported results in this study are not publicly available due to privacy restrictions.

## References

[1] Xu J., Li Z., Zhang K., Yang J., Gao N., Zhang Z., Meng Z. (2023). The Principle, Methods and Recent Progress in RFID Positioning Techniques: A Review. IEEE J. Radio Freq. Identif..

[2] Xie L., Li Q., Chen X., Lu S., Chen D. Continuous Scanning with Mobile Reader in RFID Systems: An Experimental Study. Proceedings of the Fourteenth ACM International Symposium on Mobile Ad Hoc Networking and Computing MobiHoc.

[3] Ding H., Qian C., Han J., Xiao J., Zhang X., Wang G., Xi W., Zhao J. (2019). Close-Proximity Detection for Hand Approaching Using Backscatter Communication. IEEE Trans. Mob. Comput..

[4] Luo J., Shin K.G. Detecting Misplaced RFID Tags on Static Shelved Items. Proceedings of the 17th Annual International Conference on Mobile Systems, Applications, and Services MobiSys.

[5] Wang P., Guo B., Wang Z., Yu Z. (2022). ShopSense: Customer Localization in Multi-Person Scenario with Passive RFID Tags. IEEE Trans. Mob. Comput..

[6] Chen X., Xie L., Wang C., Lu S. Adaptive Accurate Indoor-Localization Using Passive RFID. Proceedings of the 2013 International Conference on Parallel and Distributed Systems ICPADS.

[7] Zhang H., Wan G.C., Tong M.S. A Dynamic Frame Slot ALOHA Algorithm Based on Deep Learning for Collision Prevention of RFID Systems. Proceedings of the 2024 Photonics & Electromagnetics Research Symposium PIERS.

[8] Tripicchio P., D’Avella S., Unetti M., Motroni A., Nepa P. (2024). A UHF Passive RFID Tag Position Estimation Approach Exploiting Mobile Robots: Phase-Only 3D Multilateration Particle Filters with No Unwrapping. IEEE Access.

[9] Gan Q. (2021). UHF RFID Technology, Products, and Applications in IoT.

[10] Xie L., Lu S. (2020). Principles, Protocols, and System Design of RFID.

[11] Deng W., Xia Y., Li Z., Pu R., Pei W. (2021). A High-Dimensional Collided Tag Quantity Estimation Method for Multi-Antenna RFID Systems. IEEE Commun. Lett..

[12] Popovski P., Fyhn K., Jacobsen R.M., Larsen T. (2011). Robust Statistical Methods for Detection of Missing RFID Tags. IEEE Wirel. Commun..

[13] Zhang J., Liu X., Chen S., Tong X., Deng Z., Gu T., Li K. (2024). Toward Robust RFID Localization via Mobile Robot. IEEE/ACM Trans. Netw..

[14] Liu X., Zhang J., Jiang S., Yang Y., Li K., Cao J., Liu J. (2021). Accurate Localization of Tagged Objects Using Mobile RFID-Augmented Robots. IEEE Trans. Mob. Comput..

[15] Chen X., Liu J., Fu C., Huang H., Sun Y.-E., Zhang X., Tao M., Zhou Z., Chen L. (2025). Advancing RFID Tag Counting with COTS Devices: The Average Time Duration Method. IEEE Trans. Netw..

[16] Bernardini F., Buffi A., Fontanelli D., Macii D., Magnago V., Marracci M., Motroni A., Nepa P., Tellini B. (2021). Robot-Based Indoor Positioning of UHF-RFID Tags: The SAR Method with Multiple Trajectories. IEEE Trans. Instrum. Meas..

[17] Cha K., Ramachandran A., Jagannathan S. Adaptive and Probabilistic Power Control Algorithms for Dense RFID Reader Network. Proceedings of the 2006 IEEE International Conference on Networking, Sensing and Control ICNSC.

[18] Gong W., Liu H., Miao X., Liu K., He W., Zhang L., Liu Y. (2016). Fast and Adaptive Continuous Scanning in Large-Scale RFID Systems. IEEE/ACM Trans. Netw..

[19] DiGiampaolo E., Martinelli F. (2018). A Robotic System for Localization of Passive UHF-RFID Tagged Objects on Shelves. IEEE Sens. J..

[20] Tripicchio P., Unetti M., D’Avella S., Buffi A., Motroni A., Bernardini F., Nepa P. (2022). A Synthetic Aperture UHF RFID Localization Method by Phase Unwrapping and Hyperbolic Intersection. IEEE Trans. Autom. Sci. Eng..

[21] Wu C., Tao B., Wu H., Gong Z., Yin Z. (2021). A UHF RFID-Based Dynamic Object Following Method for a Mobile Robot Using Phase Difference Information. IEEE Trans. Instrum. Meas..

[22] Xie Y., Gu T., Zheng D., Zhang Y., Huan H. (2023). A High-Precision 3D Target Perception Algorithm Based on a Mobile RFID Reader and Double Tags. Remote Sens..

[23] Xi Z., Liu X., Luo J., Zhang S., Guo S. (2021). Fast and Reliable Dynamic Tag Estimation in Large-Scale RFID Systems. IEEE Internet Things J..

[24] Chen H., Xue G., Wang Z. (2017). Efficient and Reliable Missing Tag Identification for Large-Scale RFID Systems with Unknown Tags. IEEE Internet Things J..

[25] Lin K., Chen H., Ai X., Shakhov V., Ni L., Yu J., Li Y. (2020). EUMD: Efficient Slot Utilization Based Missing Tag Detection with Unknown Tags. J. Netw. Comput. Appl..

[26] Wang T., Wang B. A Cardinality Estimation Scheme for the Number of Unknown RFID Tags Under Unreliable Channels. Proceedings of the 2022 IEEE International Conference on Smart Internet of Things SmartIoT.

[27] Su J., Sheng Z., Liu A.X., Han Y., Chen Y. (2022). Capture-Aware Identification of Mobile RFID Tags with Unreliable Channels. IEEE Trans. Mob. Comput..

[28] Rodić L.D., Stančić I., Zovko K., Perković T., Šolić P. (2022). Tag Estimation Method for ALOHA RFID System Based on Machine Learning Classifiers. Electronics.

[29] Wang H., Gong W. (2021). RF-Pen: Practical Real-Time RFID Tracking in the Air. IEEE Trans. Mob. Comput..

[30] Han H., Sheng B., Tan C.C., Li Q., Mao W., Lu S. Counting RFID Tags Efficiently and Anonymously. Proceedings of the 2010 Proceedings IEEE INFOCOM.

